# Healthy aging: Linking causal mechanisms with holistic outcomes

**DOI:** 10.1111/acel.14065

**Published:** 2023-12-18

**Authors:** Monty Montano, Krisann K. Oursler, Vincent C. Marconi

**Affiliations:** ^1^ Department of Medicine Harvard Medical School Boston Massachusetts USA; ^2^ Department of Medicine Virginia Tech Carilion School of Medicine Roanoke Virginia USA; ^3^ Salem Veterans Affairs Health Care System Salem Virginia USA; ^4^ Atlanta Veterans Affairs Health Care System Decatur Georgia USA; ^5^ Hubert Department of Global Health, Rollins School of Public Health Emory University Atlanta Georgia USA; ^6^ Division of Infectious Diseases Emory University School of Medicine Atlanta Georgia USA; ^7^ Emory Vaccine Center Atlanta Georgia USA

## Abstract

Identifying and understanding the impact of differing exposures over the lifecourse necessitates contextualizing different levels of influence ranging from genetics, epigenetics, geography, and psychosocial networks.

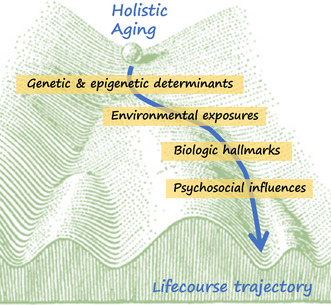

**Abbreviations**
CANTOS: canakinumab anti‐inflammatory thrombosis outcomes study
CD8+T‐cells: cluster of differentiation 8+ cytotoxic T‐cell
COVID: coronavirus disease 2019
CRF: cardiorespiratory fitness
HIV: human immunodeficiency virus
IL‐1β: interleukin 1 beta
NAD: nicotinamide adenine dinucleotide
NCCIH: National Center for Complementary and Integrative Health
SASP: senescence‐associated secretory phenotype

## 1 MOTIVATION FOR THIS SPECIAL ISSUE

Over the last 50 years, there has been remarkable progress in our understanding of cellular and molecular mechanisms that influence biological aging, with the promise of improving not only lifespan but also healthspan. However, because healthspan is affected by diverse environmental exposures (both biological and psychosocial) and genetic heterogeneity, applying this progress at a personalized level remains challenging.

Identifying and understanding the impact of differing exposures over the lifecourse (both beneficial and detrimental) necessitates contextualizing different hierarchical levels of influence ranging from geography to society to personal networks across a wide range of domains, including ecological (e.g., air pollution and clean water), psychosocial (e.g., structural disparities and stress response) and lifestyle (e.g., diet, exercise, and biobehavioral patterns).

Approaching aging as a contextual regulation and inter‐connectedness within a whole‐body framework (i.e., holistic) may provide a path toward personalizing geroscience. Further to this point, the editorial by Ferrucci et al. (2023) in this special issue calls for a collaboration between gerontologists and geriatricians, to better assess personalized health trajectories that promote healthy aging.

This thematic issue was motivated by the idea that there is value in framing healthy aging as a holistic outcome requiring whole‐body approaches and invited manuscripts along three interconnected themes: (1) biological pathways, (2) holistic approaches, and (3) interventions targeting multiple organ systems and mechanistic pathways.

## 2 BIOLOGICAL PATHWAYS

Central to geroscience is the quest to identify and understand biological mechanisms (i.e., hallmarks) that increase disease risk with chronological aging. As organisms age, damage accumulation and biological entropy increase susceptibility to morbidity, including cancer, heart disease, neurodegenerative disorders, and frailty.

A prominent hallmark of aging is the accumulation of cells with a senescent phenotype. First described in the early 1960s, senescent cells were initially considered a natural defense against cancer, as they prevent damaged cells from proliferating. However, over time, their accumulation is now recognized to contribute to age‐related tissue deterioration and chronic diseases, highlighting their paradoxical role in both promoting and impeding healthy aging. In their review, Kuehnemann & Wiley et al. (2023) discuss senescent cells and this dual nature, highlighting the contribution of senescent cells to aging and disease alongside their essential role in tissue homeostasis, and underscores the necessity of targeted interventions to mitigate their negative effects while preserving their beneficial aspects to improve age‐related outcomes.

Nicotinamide adenine dinucleotide (NAD) is a critical coenzyme in cellular metabolism, playing a key role in aging and tissue homeostasis. NAD levels decline with age, contributing to the deterioration of a range of cellular processes, notably mitochondrial function. Claudia Chini and colleagues review NAD metabolism in a context of senescence and healthy aging (Chini et al., (2023), highlighting the potential of NAD replacement therapies and their interaction with other aging hallmarks, suggesting a comprehensive understanding of these interactions is crucial for advancing age‐related disease treatments consistent with the geroscience hypothesis.

Centenarians hold a special fascination among gerontologists and the popular press. However, discovering and deciphering the role of genetic variants in centenarians that confer protection from age‐related diseases and contribute to longevity remains a challenge. In a study by Wang et al. (2023), fewer somatic mutations in centenarians were observed when compared to younger controls, revealing specific genomic regions conserved in these long‐lived individuals, alongside an enhanced DNA repair ability, highlighting these factors as potentially crucial for human longevity.

The African killifish has emerged as a valuable model organism; with a lifespan of just a few months, it is uniquely suited for studying the biological processes of aging and age‐related diseases. Its genome has also been sequenced, further allowing for genetic manipulation and association studies. Ruparelia et al. (2023) describe the utility of this model for studying sarcopenia and longevity, showing age‐related muscle function decline and notable reversals to “earlier‐life” phenotypes in older age. In their study, triglyceride depletion was used to improve lipid metabolism and nutrient homeostasis through SIRT1 as a model for calorie restriction.

Understanding organ crosstalk is vital for developing holistic and integrative approaches to enhance healthspan and to potentially extend lifespan. Moaddel and colleagues (2023) conducted a cross‐sectional analysis of healthy individuals aged 22–92 years old to provide a comprehensive assessment of age‐related biomarkers across different biofluids and tissues within the same individuals, revealing interconnected age‐associated metabolic pathways to facilitate the discovery of mechanisms underlying phenotypic aging.

The significance of cardiorespiratory fitness (CRF) in healthspan and lifespan has been increasingly recognized over the past few decades. CRF refers to the ability of the cardiovascular and respiratory systems to supply oxygen to skeletal muscles for cellular respiration and bioenergetics during sustained physical activity. The link between CRF and DNA methylation clocks is also an emerging area of research, bridging physical fitness with molecular biomarkers that measure the rate or pace of aging. Kawamura et al. (2023) describe associations between CRF and lifestyle‐related factors with DNA methylation in a cohort of older men, finding that higher CRF levels correlated with delayed biological aging, though lifestyle factors (e.g., smoking) had a more significant impact, highlighting the nuanced interplay between fitness, behavioral exposures, and biological aging.

## 3 HOLISTIC APPROACHES

A perspective by Langevin et al. (2023) define healthy aging as an integrated, whole‐body process that is based on biology, behavior, and the social/physical environment. From the vantage point of the National Center for Complimentary and Integrative Health (NCCIH), this commentary describes the challenges to integrated, multicomponent interventions for healthy aging and advocate for a balanced exploration of pharmacologic and nonpharmacologic strategies.

Development of holistic outcomes that gauge both the pathogenetic process and end‐organ damage is necessary to investigate prevention and intervention strategies at the whole‐body level. Jiang et al. (2023) describe a cross‐sectional study in older adults with and without hypertension wherein they examine the association between the complexity of beat‐to‐beat blood pressure fluctuations and vascular alterations due to aging. Lower complexity correlated with greater arterial stiffness and worse endothelial function. Additional multimodal strategies that counteract physiological decline and multisystem dysregulation through movement‐based mind–body therapies were reviewed by Loewenthal et al. (2023), with examples of the potential for Tai Chi and yoga as preventative strategies to treat frailty in older adults. Ma et al. (2023) provided further discussion of this approach in a study comparing long‐term and short‐term Tai Chi practice in older adults.

Acosta et al. (2023) provide an innovative commentary on the value of integrating humanism into patient care to address both physical and metaphysical needs. Notably, they point out associations of spirituality with long‐term health outcomes that would not have been considered in more mechanistically driven studies. This point is further exemplified in a study of bereavement by Palitsky et al. (2023) that demonstrates how religious and existential variables interplay with psychosocial factors and biomarkers related to cardiovascular risk.

## 4 AGING INTERVENTIONS

Identifying efficacious interventions that are broadly acceptable, cost‐effective, and scalable remains the coin of the realm for geroscientists. In this special issue, Li and colleagues (2023) suggest a more nuanced strategy for designing interventions aimed at promoting healthy aging. Instead of targeting a specific aging hallmark with associated phenotypes, pathways effecting multiple hallmarks of aging should be targeted (e.g., mitochondrial dysfunction), positing that interventions targeting key hallmarks could have cascading effects across the aging process.

First described over 100 years ago, metformin has become the most widely prescribed oral medication for diabetes mellitus and is experiencing a rediscovery of sorts for other indications including cancer, tuberculosis, weight loss, COVID‐19, and longevity. Corley et al. (2023) examined the impact of metformin on epigenetics in immune aging of monocytes and CD8+ T cells from virally suppressed older adults with HIV infection. This study identified cell‐type‐specific myeloid effects, as evidenced by decreased epigenetic age in monocytes, but without a significant change in CD8+ T cells. Given the differing geroprotective outcomes associated with metformin treatment (e.g., Bannister et al., 2014; Keys et al., 2022), large clinical trials using diverse populations are encouraged to better interpret heterogeneity.

Although canakinumab, a monoclonal antibody inhibiting IL‐1β, reduces age‐related diseases including major adverse cardiovascular events, non‐small cell lung cancer, inflammatory anemia, gout, and large joint osteoarthritis, a role for canakinumab in frailty outcomes is unclear. In this issue, a study of more than 10,000 participants enrolled in the CANTOS trial is described. The individuals in this cohort had a previous myocardial infarction, and notably frailty, measured as a 34‐item cumulative deficit index, was not reduced, despite reductions in the inflammatory biomarker C‐reactive protein (Orkaby et al., 2023). The results highlight the elusive nature of the frailty phenotype when approached from a system‐based manner, highlighting the need for a whole‐body framework.

There are several natural products with senomorphic properties including polyphenols and flavonoids. Here, Liu et al. (2023) screened a natural medicinal agent library and describe rutin, a phytochemical with potent senomorphic activity against senescent cells and the senescence‐associated secretory phenotype (SASP), as well as improving the efficacy of chemotherapeutic treatments.

Finally, this special issue highlighted the benefits of behavioral interventions that target physical activity and exercise. Two of the three studies report on the impact of exercising on human skeletal muscle. Voisin et al. (2023) conducted an extensive meta‐analysis of 3176 specimens including longitudinal cohorts of individuals exercising (aerobic, resistance, or high‐intensity interval training) and those experiencing muscle disuse. This study revealed that higher baseline aerobic fitness correlated with younger epigenetic and transcriptomic profiles, and muscle disuse resulted in aging of the transcriptome. In a second study by Jankowski et al. (2023), epigenetic age acceleration and DNA methylation markers in skeletal muscle were measured in older adults with and without HIV, before and after a 24‐week exercise program. A third study by Manning et al. (2023) compared the results of a physical performance battery among sedentary Italian adults (Act on Ageing) and U.S. Veterans participating in an exercise program (Gerofit). These studies collectively provide further evidence that exercise training is a viable strategy to decelerate or reverse molecular aging, particularly in functional capacity and aging in humans.

## 5 THE ROAD AHEAD

Although lifespan has dramatically improved over the past 70 years, multiple age‐related diseases have nevertheless increased and have been exacerbated by sedentary lifestyles, psychological stress, processed foods, and weakened social networks. Furthermore, low‐ and middle‐income populations and regions have not been as fortunate to realize all of these benefits especially for specific diseases such as diabetes and obesity (Safiri et al., 2022).

Additionally, polypharmacy associated with treatment of individuals having multi‐morbidity is not without a cost and has been independently associated with early mortality (Justice et al., 2018). Finally, aging in the modern world has become increasingly complex as cognitive decline, isolation, and social fragmentation have left many individuals feeling abandoned within our increasingly digital environment.

In this special issue on healthy aging, we chose to emphasize the multidimensional aspects of aging and the need for transdisciplinary research and intervention strategies. This special issue underscores the importance of understanding the mechanistic links between psychosocial experiences and biological markers, delving into the phenotypes of biological aging, and advocating for physical activity and exercise training as pivotal interventions for promoting healthy aging. Inspired by Patrick Pietroni's definition of the patient as a holistic being (Pietroni, 1997), this issue emphasizes that healthy aging transcends the mere absence of disease, requiring an integrated strategy that addresses the cognitive, behavioral, motor, sensory, autonomic, cellular, molecular, and affective domains to optimize the lived experience.

Several goalposts were identified from this special issue. First, composite markers that capture both global senescence and organ‐specific aging phenotypes need to be developed, engaging next‐generation analytical methodologies that incorporate functional assessments, multi‐morbidity, and biological profiles, capturing both positive and negative effects of environmental exposure across the entire lifespan. These markers should also strike a balance between population‐based public health approaches and personalized medical care by providing access to precision medicine for all individuals regardless of income or geography. Second, this issue encourages the implementation of proof‐of‐concept trials targeting multiple mechanistic pathways of aging. This will require collaboration across disciplines and scientific perspectives and will challenge the existing funding structures, urging for updates to accommodate interdisciplinary research in aging, with the goal of prioritizing healthspan over lifespan.

Finally, it is critical that this field not undervalue features of aging such as dignity, beauty, and subjective appreciation of life. We must provide a respectful, supportive, and caring environment for older adults where their experiences are heard, their wisdom is valued, and their presence is cherished.

## 6 AUTHOR CONTRIBUTIONS

MM initially conceived of special issue topic. MM, VCM, and KAO wrote, revised, and approved final manuscript. VCM and KAO served as guest editors and assisted in recruiting authors.

## FUNDING INFORMATION

VCM has received investigator‐initiated research grants (to the institution) and consultation fees from Eli Lilly, Bayer, Gilead Sciences, Merck, and ViiV. VCM has received funding support from NIH/NIDDK (R01DK125187) and the Emory Center for AIDS Research (P30AI050409) for work related to this manuscript. MM acknowledges support from the Boston Claude D. Pepper OAIC (P30AG031679).

## CONFLICT OF INTEREST STATEMENT

No conflicts to declare.

## Data Availability

Data sharing is not applicable to this article as no new data were created or analyzed in this study.

## References

[acel14065-bib-1001] Acosta, L. M. Y. , & Ely, E. W. (2023). Holistic care in healthy aging: Caring for the wholly and holy human. Aging Cell, 23, e14021. 10.1111/acel.14021 37873723 PMC10776114

[acel14065-bib-0001] Bannister, C. A. , Holden, S. E. , Jenkins‐Jones, S. , Morgan, C. L. , Halcox, J. P. , Schernthaner, G. , Mukherjee, J. , & Currie, C. J. (2014). Can people with type 2 diabetes live longer than those without? A comparison of mortality in people initiated with metformin or sulphonylurea monotherapy and matched, non‐diabetic controls. Diabetes, Obesity & Metabolism, 16(11), 1165–1173. 10.1111/dom.12354 25041462

[acel14065-bib-1002] Chini, C. C. S. , Cordeiro, H. S. , Tran, N. L. K. , & Chini, E. N. (2023). NAD metabolism: Role in senescence regulation and aging. Aging Cell, 23, e13920. 10.1111/acel.13920 37424179 PMC10776128

[acel14065-bib-1003] Corley, M. J. , Pang, A. P. S. , Shikuma, C. M. , & Ndhlovu, L. C. (2023). Cell‐type specific impact of metformin on monocyte epigenetic age reversal in virally suppressed older people living with HIV. Aging Cell, 23, e13926. 10.1111/acel.13926 37675817 PMC10776116

[acel14065-bib-1004] Ferrucci, L. , Wilson, D. M. , Donega, S. , & Montano, M. (2023). Enabling translational geroscience by broadening the scope of geriatric care. Aging Cell, 23, e14034. 10.1111/acel.14034 38038340 PMC10776120

[acel14065-bib-1005] Jankowski, C. M. , Konigsberg, I. R. , Wilson, M. P. , Sun, J. , Brown, T. T. , Julian, C. G. , & Erlandson, K. M. (2023). Skeletal muscle DNA methylation: Effects of exercise and HIV. Aging Cell, 23, e14025. 10.1111/acel.14025 37920126 PMC10776118

[acel14065-bib-1006] Jiang, X. , Mang, X. , Zhou, H. , Chen, J. , Tan, H. , Ren, H. , Huang, B. , Zhong, L. , Lipsitz, L. A. , Manor, B. , Guo, Y. , & Zhou, J. (2023). The physiologic complexity of beat‐to‐beat blood pressure is associated with age‐related alterations in blood pressure regulation. Aging Cell, 23, e13943. 10.1111/acel.13943 37615223 PMC10776119

[acel14065-bib-0002] Justice, A. C. , Gordon, K. S. , Skanderson, M. , Edelman, E. J. , Akgun, K. M. , Gibert, C. L. , Lo Re, V., 3rd , Rimland, D. , Womack, J. A. , Wyatt, C. M. , Tate, J. P. , & VACS Project Team . (2018). Nonantiretroviral polypharmacy and adverse health outcomes among HIV‐infected and uninfected individuals. AIDS, 32(6), 739–749. 10.1097/QAD.0000000000001756 29543653 PMC5868488

[acel14065-bib-1007] Kawamura, T. , Radak, Z. , Tabata, H. , Akiyama, H. , Nakamura, N. , Kawakami, R. , Ito, T. , Usui, C. , Jokai, M. , Torma, F. , Kim, H. , Miyachi, M. , Torii, S. , Suzuki, K. , Ishii, K. , Sakamoto, S. , Oka, K. , Higuchi, M. , Muraoka, I. , … Tanisawa, K. (2023). Associations between cardiorespiratory fitness and lifestyle‐related factors with DNA methylation‐based ageing clocks in older men: WASEDA'S Health Study. Aging Cell, 23, e13960. 10.1111/acel.13960 37584423 PMC10776125

[acel14065-bib-0003] Keys, M. T. , Thinggaard, M. , Larsen, L. A. , Pedersen, D. A. , Hallas, J. , & Christensen, K. (2022). Reassessing the evidence of a survival advantage in type 2 diabetes treated with metformin compared with controls without diabetes: A retrospective cohort study. International Journal of Epidemiology, 51(6), 1886–1898. 10.1093/ije/dyac200 36287641

[acel14065-bib-1008] Kuehnemann, C. , & Wiley, C. D. (2023). Senescent cells at the crossroads of aging, disease, and tissue homeostasis. Aging Cell, 23, e13988. 10.1111/acel.13988 37731189 PMC10776127

[acel14065-bib-1009] Langevin, H. M. , Weber, W. , & Chen, W. (2023). Integrated multicomponent interventions to support healthy aging of the whole person. Aging Cell, 23, e14001. 10.1111/acel.14001 37840416 PMC10776112

[acel14065-bib-1010] Li, Y. , Berliocchi, L. , Li, Z. , & Rasmussen, L. J. (2023). Interactions between mitochondrial dysfunction and other hallmarks of aging: Paving a path toward interventions that promote healthy old age. Aging Cell, 23, e13942. 10.1111/acel.13942 37497653 PMC10776122

[acel14065-bib-1011] Liu, H. , Xu, Q. , Wufuer, H. , Li, Z. , Sun, R. , Jiang, Z. , Dou, X. , Fu, Q. , Campisi, J. , & Sun, Y. (2023). Rutin is a potent senomorphic agent to target senescent cells and can improve chemotherapeutic efficacy. Aging Cell, 23, e13921. 10.1111/acel.13921 37475161 PMC10776113

[acel14065-bib-1012] Loewenthal, J. , Berning, M. J. , Wayne, P. M. , Eckstrom, E. , & Orkaby, A. R. (2023). Holistic frailty prevention: The promise of movement‐based mind–body therapies. Aging Cell, 23, e13986. 10.1111/acel.13986 37698149 PMC10776124

[acel14065-bib-1013] Ma, Y. , Gow, B. J. , Song, R. , Rist, P. M. , Hausdorff, J. M. , Lipsitz, L. A. , Manor, B. , & Wayne, P. M. (2023). Long‐term Tai Chi practice in older adults is associated with “younger” functional abilities. Aging Cell, 23, e14023. 10.1111/acel.14023 37905388 PMC10776109

[acel14065-bib-1014] Manning, K. M. , Hall, K. S. , Sloane, R. , Magistro, D. , Rabaglietti, E. , Lee, C. C. , Castle, S. , Kopp, T. , Giffuni, J. , Katzel, L. , McDonald, M. , Miyamoto, M. , Pearson, M. , Jennings, S. C. , Bettger, J. P. , & Morey, M. C. (2023). Longitudinal analysis of physical function in older adults: The effects of physical inactivity and exercise training. Aging Cell, 23, e13987. 10.1111/acel.13987 37681737 PMC10776115

[acel14065-bib-1015] Moaddel, R. , Ubaida‐Mohien, C. , Tanaka, T. , Tian, Q. , Candia, J. , Moore, A. Z. , Lovett, J. , Fantoni, G. , Shehadeh, N. , Turek, L. , Collingham, V. , Kaileh, M. , Chia, C. W. , Sen, R. , Egan, J. M. , & Ferrucci, L. (2023). Cross‐sectional analysis of healthy individuals across decades: Aging signatures across multiple physiological compartments. Aging Cell, 23, e13902. 10.1111/acel.13902 37350292 PMC10776121

[acel14065-bib-1016] Orkaby, A. R. , Thomson, A. , MacFadyen, J. , Besdine, R. , Forman, D. E. , Travison, T. G. , & Ridker, P. M. (2023). Effect of canakinumab on frailty: A post hoc analysis of the CANTOS trial. Aging Cell, 23, e14029. 10.1111/acel.14029 37927208 PMC10776110

[acel14065-bib-1017] Palitsky, R. , Chen, Z. J. , Rentscher, K. E. , Friedman, S. E. , Wilson, D. M. T. , Ruiz, J. M. , Sullivan, D. , Grant, G. H. , & O'Connor, M. (2023). Associations of religious and existential variables with psychosocial factors and biomarkers of cardiovascular risk in bereavement. Aging Cell, 23, e14014. 10.1111/acel.14014 37840393 PMC10776136

[acel14065-bib-0004] Pietroni, P. (1997). Is complementary medicine holistic? Complementary Therapies in Nursing & Midwifery, 3(1), 9–11. 10.1016/s1353-6117(97)80027-3 9432425

[acel14065-bib-1018] Ruparelia, A. A. , Salavaty, A. , Barlow, C. K. , Lu, Y. , Sonntag, C. , Hersey, L. , Eramo, M. J. , Krug, J. , Reuter, H. , Schittenhelm, R. B. , Ramialison, M. , Cox, A. , Ryan, M. T. , Creek, D. J. , Englert, C. , & Currie, P. D. (2023). The African killifish: A short‐lived vertebrate model to study the biology of sarcopenia and longevity. Aging Cell, 23, e13862. 10.1111/acel.13862 37183563 PMC10776123

[acel14065-bib-0005] Safiri, S. , Karamzad, N. , Kaufman, J. S. , Bell, A. W. , Nejadghaderi, S. A. , Sullman, M. J. M. , Moradi‐Lakeh, M. , Collins, G. , & Kolahi, A. A. (2022). Prevalence, deaths and disability‐adjusted‐life‐years (DALYs) due to type 2 diabetes and its attributable risk factors in 204 countries and territories, 1990‐2019: Results from the global burden of disease study 2019. Front Endocrinol (Lausanne), 13, 838027. 10.3389/fendo.2022.838027 35282442 PMC8915203

[acel14065-bib-1019] Voisin, S. , Seale, K. , Jacques, M. , Landen, S. , Harvey, N. R. , Haupt, L. M. , Griffiths, L. R. , Ashton, K. J. , Coffey, V. G. , Thompson, J. M. , Doering, T. M. , Lindholm, M. E. , Walsh, C. , Davison, G. , Irwin, R. , McBride, C. , Hansson, O. , Asplund, O. , Heikkinen, A. E. , … Eynon, N. (2023). Exercise is associated with younger methylome and transcriptome profiles in human skeletal muscle. Aging Cell, 23, e13859. 10.1111/acel.13859 37128843 PMC10776126

[acel14065-bib-1020] Wang, H. , Zhao, L. , Yang, L. , Ge, M. , Yang, X. , Gao, Z. , Cun, Y. , Xiao, F. , & Kong, Q. (2023). Scrutiny of genome‐wide somatic mutation profiles in centenarians identifies the key genomic regions for human longevity. Aging Cell, 23, e13916. 10.1111/acel.13916 37400997 PMC10776117

